# Structure and Mechanical Properties of High-Density Polyethylene Composites Reinforced with Glassy Carbon

**DOI:** 10.3390/ma14144024

**Published:** 2021-07-19

**Authors:** Piotr Olesik, Marcin Godzierz, Mateusz Kozioł, Jakub Jała, Urszula Szeluga, Jerzy Myalski

**Affiliations:** 1 Faculty of Materials Engineering, Silesian University of Technology, Krasińskiego 8 Street, 40-019 Katowice, Poland; Piotr.olesik@polsl.pl (P.O.); mgodzierz@cmpw-pan.edu.pl (M.G.); jakjala97@gmail.com (J.J.); jrezy.myalski@polsl.pl (J.M.); 2 Centre of Polymer and Carbon Materials, Polish Academy of Sciences, M. Curie-Skłodowskiej 34 Street, 41-819 Zabrze, Poland; uszeluga@cmpw-pan.edu.pl

**Keywords:** high-density polyethylene (HDPE), polymer matrix composite, glassy carbon, X-ray diffraction, residual stress analysis

## Abstract

In this paper, we investigated theimpact of glassy carbon (GC) reinforcement oncrystal structure and the mechanical performance of high-density polyethylene (HDPE). We made composite samples by mixing HDPE granules with powder in ethanol followed bymelt mixing in a laboratory extruder. Along with the investigated composite, we also prepared samples with carbon nanotubes (CNT), graphene (GNP) and graphite (Gr) to compare GC impact with already used carbon fillers. To evaluate crystal structure and crystallinity, we used X-ray diffraction (XRD) and differential scanning calorimetry (DSC). We supported the XRD results with a residual stress analysis (RSA) according to the EN15305 standard. Analysis showed that reinforcing with GC leads to significant crystallite size reduction and low residual stress values. We evaluated the mechanical properties of composites with hardness and tensile testing. The addition of glassy carbon results inincreased mechanical strength incomposites with CNT and GNP.

## 1. Introduction

High-density polyethylene (HDPE) is one of the most used thermoplastics, due to its high availability, low cost and superior processing properties [1]. Industrial applications of HDPE include toys, utensils, films, bottles, pipes and processing equipment as well as wire and cable insulations. Due to its chemical resistance and bio-neutrality, HDPE may also be used as a prosthesis acetabulum material in various prostheses. It is mainly used when considering materials for hip prostheses. Although ultra-high molecular weight polyethylene (UHMWPE) is primarily utilized in hip prostheses, HDPE exhibits much better processing properties even though it has slightly worse tribological properties. For this reason, HDPE remains a competitive material in this group of applications [2,3,4,5,6].

Literature reports the effect on the crystallization process and material properties when carbon fillers are introduced into polyolefins. Fouad et al. [6] proved that the addition of 4 wt% of nano-graphite increased the crystallinity of the HDPE matrix while simultaneously increasing the mechanical properties of the composite. However, a strong decrease in the plastic properties of the material was also detected. Dabees et al. [7] denoted an increase in hardness, toughness, and tensile strength of MWCNT-modified HDPE even if only 1 wt% of CNT was added. Moreover, they detected lower water absorption and higher ductility during tensile tests of CNT-modified composites in comparison to the neat polymer. Pelto et al. [8] described the effect of the application of 1 wt% of graphene oxide (GO), which induces additional crystallization, which in turn causes crystallite refinement. Similar results were described by Weng [9], but for a relatively high amount (10–40 wt%) of expanded graphite. Sahu et al. [10] described a positive effect of a relatively low amount (up to 3 wt%) of carbon black on the crystallinity and UV resistance of HDPE composites. In contrast to previous studies, Gaska [11] and Paszkiewicz [12] described the negative effect of MWCNT, GNP, and the introduction of these two materials into a low-density polyethylene (LDPE) matrix on the crystallinity of the polymer. However, they proved that relatively high amounts (above 5 wt%) of carbon nanoparticles strongly impeded the crystallization of the polymer. In addition to an increase in tensile strength in the examined composites, which was proven in both papers, an increase in the mechanical properties was also seen when graphene nanoplatelets were introduced into LDPE. Moreover, literature reports [13] an increase in melt viscosity with the addition of higher amounts of graphene nanoplatelets. The addition of nanometric carbon reinforcement in polypropylene impedes crystallization of β-modification while simultaneously increasing the crystallinity of the polymer because of the higher amount of α-modification [11,13,14]. Combined, these effects lead to a slightly higher strength nanocomposite with a highly limited elastic deformation of the material [15]. In contrast, the addition of 1 wt% of graphene oxide (GO) in a polyamide 6 (PA6) matrix favors crystallization of γ-modification of PA6 [16]. However, there is a large difference in the chain structure of polypropylene (PP) and PA6, which could impede proper comparison. The introduction of carbon nanoparticles into polyolefins has not only a positive impact on the mechanical performance of polyolefins but also on the tribological properties. Xu et al. [17,18] proved that the addition of 3 wt% carbon nanofibers (CNF) reduces the coefficient of friction in contact with a steel counter sample drastically, while the wear rate increases. However, the introduction of lower amounts of CNF (0.5 and 1 wt%) increases the coefficient of friction but simultaneously decreases the wear rate. A similar effect was obtained by Liu [19] for 3 wt% of silanized GNPs-filled HDPE.The above nano-reinforcements haveavariety of properties;however, a technological challenge limits their application. As a different carbon filler, glassy carbon can be used.

A glassy carbon (GC) is a form of carbon that consists of distorted graphene layers in a structure. Such material is obtained with the pyrolysis of phenylic resins. Glassy carbon is mostly used as electrodes in chemistry due to its stability. However, it has been reported recently as a possible reinforcement for thermoplastic composites. In our previous work [16], we presented a PA6/GC composite with improved wear resistance. Additionally, it shows that GC does not tend to agglomerate in melted plastic, which makes glassy carbon less technologically challenging than nanocarbon fillers.

In this paper we present a novel HDPE-based composite reinforced with glassy carbon fine particles. We report a wide study of GC impact on crystallization, crystal structure and mechanical properties. An important part of the study is the determination of the residual stress of crystal structure according to EN15305 standard, which was made for the first time for polymer materials. We evaluated the obtained HDPE/GC composites, comparing them to composites with graphite, carbon nanotubes and graphene.

## 2. Materials and Methods

### 2.1. Materials for Research and Composites Fabrication Method

As the matrix for the composites, we used HDPE Hivorex 2600J (by Lotte Chemical, Seoul, Korea). We obtained glassy carbon powder by high-energy milling at a planetary ball mill glassy carbon foams made by Prof. Jerzy Myalski (Silesian University of Technology, Faculty of Materials Engineering, Poland). The GC powder mean diameter D [3:2] was measured with a Malvern MasterSizer3000 (MalvernPanalytical, Malvern, UK) as 4.19 µm.

To make composites for comparison with HDPE/GC we used carbon nanotubes, graphene and graphite powders. The carbon nanotubes were supplied from Sigma Aldrich with mean diameters of 6–9 nm and 5 µm length. The graphite powder was supplied from Merck with a powder mean diameter of <50 µm. The graphene nanopowder was supplied from Sigma Aldrich with a specific surface area of 500 m^2^/g.

First, we deagglomerated the carbon reinforcement in ethyl alcohol for 1 h in an ultrasonic bath (SONIC-3 by Polsonic, Warsaw, Poland, 750 W). Then we mixed each reinforcement powder with the HDPE granules by ultrasonic treatment in ethyl alcohol as the dispersion liquid. The ultrasonic treatment was carried out for 30 min followed bymechanical stirring at 80 °C until all alcohol evaporated. This process concluded with covering granules with a thin layer of reinforcement powder (pre-composite granules). In each case, the amount of the powder was fixed at 5 g per 150 g of HDPE.

Next, we melt mixed each composite using ZAMAK DTR EHP-2 × 16S twin screw extruder (by Zamak, Skawina, Poland). The obtained composite extrudates were then cut into pieces and re-melted in a steel mold (inner dimensions 150 mm × 50 mm × 2.5 mm) at 200 °C in a furnace (ELF 11/14B by Carbolite, Hope Valley, UK) to obtain equal cooling conditions. The cooling rate was 1 °C/min. All samples for tests were cut from the obtained panels.

### 2.2. Examination Methods

We characterized the melting and crystallization behavior of HDPE and the composites using a differential scanning calorimeter (DSC 2920, TA Instruments, New Castle, DE, USA) under a nitrogen atmosphere with a nitrogen flow rate of 50 mL/min. The non-modulated DSC used in this study leads to less accurate values of the crystallinity degree compared to values obtained with modulated DSC. However, the performed baseline calibration in the temperature ranges from 0 to 300 °C and the calibration with the indium standard, as well as the exclusion of additional crystallization effects for the tested HDPE samples at the cooling rate of 5 °C/min allow us to consider this method as relatively correct. Samples of approximately 10 mg were encapsulated in standard non-hermetic aluminum pans and were heated from room temperature to 200 °C at a rate of 10 °C/min. After melting, the samples were held in the DSC chamber for 5 min to eliminate the thermal history of the samples before being cooled to room temperature at a rate of 10 °C/min using a liquid nitrogen cooling accessory. From this data, we determined the crystallization temperature (T_c_) and the crystallization enthalpy (ΔH_c_). After completing the first complex melt-crystallization thermograms, samples were heated in the second cycle from room temperature to 200 °C. The melting temperature (T_m_) and the melting enthalpy (heat of fusion, ΔH_m_) were determined in accordance with ISO 11357-3 standard.

We calculated the degree of crystallinity (X_c_(%)) for pure HDPE and the composites using Equation (1) [20,21,22,23]:

(1) $$X_{c}\left(\%\right)=\frac{∆H_{m}}{∆H_{m}^{0}\times \left(1-\varphi \right)}\times 100$$

where ΔH_m_ is the experimental melting enthalpy value of sample (J/g) obtained in the second heating DSC cycle, φ is the mass fraction of the filler, the $∆H_{m}^{0}$ is the melting enthalpy of 100% perfectly crystalline form of HDPE (293 J/g) [24,25,26].

We performed the XRD analysis using the D8 Advance diffractometer (Bruker, Karlsruhe, Germany) with Cu-Kα cathode (λ = 1.54 nm). The scan rate was 0.25°/min with scanning step of 0.02° in the range of 5° to 60° 2Θ. To identify the fitting phases, we used DIFFRAC.EVA V5.1program with ICDD PDF#2 database. We also calculated crystalline size, lattice strain and lattice parameters of Pbnm orthorhombic HDPE using Rietveld refinement in the TOPAS 6 program, which is based on Williamson-Hall theory. We used the pseudo-Voigt function in the description of the diffraction line profiles in the Rietveld refinement. To evaluate thequality of fitting experimental data to calculations we used theRwp (weighted-pattern factor) and S (goodness-of-fit) parameters. All measurements and analyses were performed three times to obtain statistically reliable results. We made residual stress analysis with the use of iso-inclination geometry. According to the EN15305 standard, the farthest possibleand single peak from the obtained diagram—the (020) peak was used for residual stress analysis of each tested sample. We evaluated the results using the LEPTOS 7 program with the following material parameters: a Young’smodulus (E) of 973 MPa and a Poisson ratio (υ) of 0.44. The value for stress-free HDPE was assumed as 0.1 MPa due to the low Young’smodulus of the polymer. To verify residual stress results, we used the extended Bragg’s law for peak position (Equation (2)) in combination with the grazing incidence XRD scan. The parts of the equation correspondingly refer to: Bragg’s law for stress free lattice, shift due to residual stress and shift due to refraction effect. The incidence angle was established as 1 degree. Using a 1-degree incidence angle eliminates the refraction effect due to critical angle (α_c_) 1°.

(2) $$2\theta_{\mathrm{hkl}}=2\mathrm{Arc}\sin\left(\frac{\lambda }{2d_{\mathrm{hkl}}^{0}}\right)+2\sigma \tan(\theta_{\mathrm{hkl}})\left(\frac{\nu +1}{E}\sin^{2}\psi -\frac{2\nu }{E}\right)+\alpha -\sqrt{\alpha^{2}-\alpha_{c}^{2}}$$

where: 2θ_hkl_—peak position, λ—wavelength of source, $d_{\mathrm{hkl}}^{0}$—interplanar spacing, σ—stress, ν—Poisson ratio, E—Young’s modulus, ψ—crystalline orientation, α—incidence angle, α_c_—critical incidence angle.

We made hardness measurements using an HK460 (Heckert, Chemnitz, Germany) device based on the Brinell method. We performed the tests under a 365 N load in accordance with the PN-EN ISO 6506-1 standard for polymer materials hardness.

We examined the tensile properties of the HDPEand its compositesafter extrusion using an Instron 4469 with 5 kN and crosshead speed of 25 mm/min, according to the PN-EN ISO 527 standard. All tests were carried out on three samples for each type of material.

## 3. Results and Discussion

### 3.1. Crystallinity Evaluations

#### 3.1.1. Wide Angle X-ray Diffraction Studies

**Figure 1:**
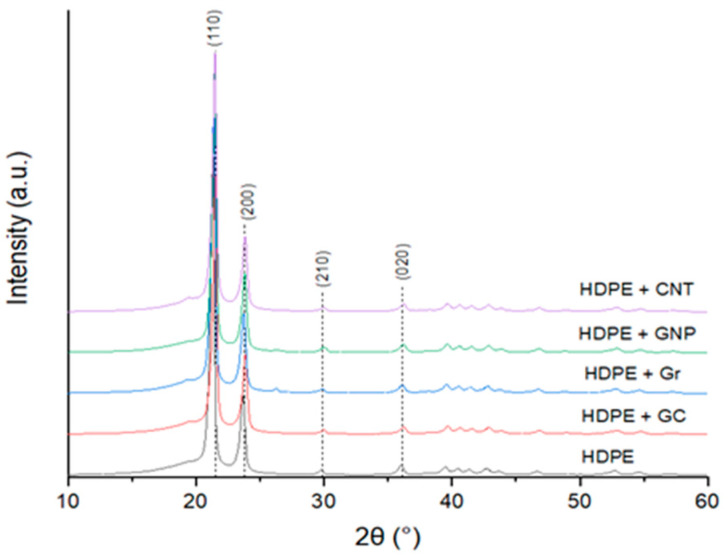
Representative WAXD pattern of neat HDPE and fabricated composites with indicated theoretical positions of (110), (200), (210) and (020) peaks.

The WAXD patterns of neat HDPE and its composites are presented in Figure 1. These figures highlight the difference in intensity and position of (110) and (200) peaks. Such phenomena correspond to residual stress present in a material, most likely as an effect of processing and fillers introduction. Considering the theoretical position of (110) and (200) peaks, it is apparent that residual stress is of tensile nature, most likely as an effect of applied manufacturing technology (extrusion followed by re-melting). However, considering the real position of (110) and (200) peaks, the introduction of carbon fillers into neat HDPE creates residual stress with a compressive nature, resulting in lower values of d-spacing (Table 1). Therefore, the introduction of carbon fillers into the HDPE matrix not only reduces crystallite size but also causes residual stress in the polymer matrix.

The crystallinity of the HDPE matrix was calculated using the peak decomposition method of WAXD patterns were in the 48–50% range. The lowest crystallinity was detected for the neat matrix, while the highest was for the GNP modification. The introduction of different carbon fillers also caused contraction of the orthorhombic lattice in comparison to the neat polymer manufactured using the same method. Volumetric contraction of lattice, calculated using Rietveld refinement, was highest for CNT and GNP modification at 1.65% and 1.63%, respectively. A slightly lower contraction was detected for GC and Gr modification (1.58%). Moreover, an applied fabrication procedure causes elongation of the lattice of up to 2.9% for neat polymer or 2.2% for composites in comparison to ICDD data (b parameters, Table 2).

#### 3.1.2. Residual Stress Analysis

Performed residual stress analysis (RSA) allows one to establish the real values of stress in materials, which is an effect of introducing fillers and applying technology (Table 2). First, it should be noted that the application of the standard RSA procedure (according to EN15305 standard) and calculation of the residual stress using the extended Bragg’s law obtains similar, comparable results in every case besides the CNT modification. To calculate stress using (020) peak, there are two parts—linear and shear stress. As it is presented in Table 2 and Table 3, for all composites, the shear part of stress was calculated in opposite to the neat polymer. The highest value of shear stress was detected for the CNT modification, which suggests major differences in comparison to the other composites. It is likely an effect of CNT agglomerates introduced into the polymer, which impede crystalline growth and cause relatively high lattice strain. The addition of graphite particles causes major compressive stress in the material, which is expected in the case of relatively big particles (around 50 μm). In the case of the introduction of GC and GNP, the tensile nature of stress was detected; however, when comparing those results with neat polymer, the addition of GC or GNP caused a slight stress reduction but remained tensile in nature. This suggests that glassy carbon has a similar impact onthecrystal structure of HDPE as graphene nanoparticles. Due to this research, we conclude that glassy carbon and graphene extend the cooling time of surrounding crystal phases in microareas. This allows stress relaxation in the structure. Glassy carbon might extend the cooling time even more due to its low thermal conductivity [27,28,29].

#### 3.1.3. Differential Scanning Calorimetry

**Figure 2:**
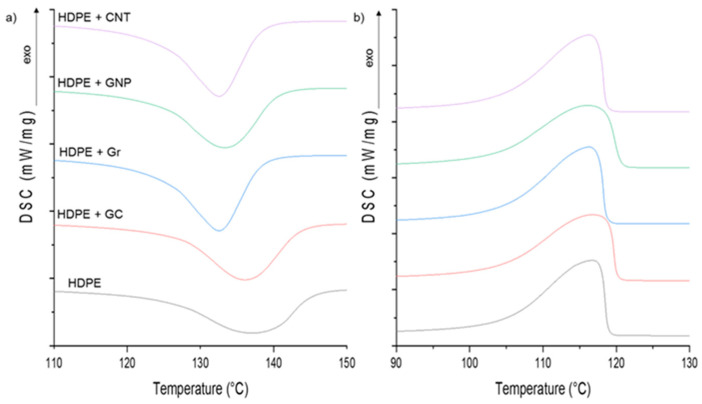
Crystallization exotherms (**a**) and melting endotherms (**b**) of HDPE and composites with various carbon fillers.

The effect of structure and properties of carbon materials used as fillers on the crystallization and melting parameters of composites based on the HDPE matrix was studied using the DSC technique. Figure 2 illustrates the melting and crystallization characteristics of HDPE and its composites.

The detailed melting temperature (T_m,max_), melting enthalpy (ΔH_m_), crystallization onset temperature (T_c,onset_), peak maximum (T_c,max_) and crystallization enthalpy (ΔH_c_) are summarized in Table 3. The crystallization peak onset and peak maximum are highest for composites with graphene nanoplatelets in comparison to pure HDPE and other composites. The increase of these temperatures is attributed to the heterogeneous nucleation induced by graphene sheets. The composite crystallization behavior could be the result of the aspect ratio of these flake fillers. This nucleating effect of graphene layers is known and has been described [22,30,31]. For other composites, the crystallization onset temperature and peak maximum are only slightly lower in comparison to values for pure HDPE. In the case of the HDPE/CNT composite, the effect might be associated with the entanglement and self-agglomeration tendencies of carbon nanotubes. However, when using the micro-sized fillers, i.e., glassy carbon and graphite, spatial hindrances during the crystallization process can affect crystallization behavior.

Analysis of the width of crystallization peaks at the base and at the half-height result in similar values, both for HDPE and for all composites, which indicates that crystallite size distribution is comparable.

It was also found that the crystallization enthalpy values for HDPE composites with graphite and graphene materials, normalized with respect to the HDPE amount, are higher than that of pure HDPE. The heat of crystallization of the HDPE/CNT composite is lower. The effects of CNTs on the crystallization behavior of polymers have been extensively studied [32,33] indicating nucleating effects of CNTs, which induce easier and faster isothermal and non-isothermal crystallization. However, in the case of the studied HDPE/CNT composite, this effect was not observed.

From the analysis of the second heating DSC runs, the melting temperature of HDPE is slightly lower as compared to all carbon/HDPE composites, while the melting enthalpy of HDPE is higher than any of the carbon/HDPE composites. Melting enthalpy is dependent on the carbon filler. The greatest reduction in ΔH_m_ value from the pure HDPE polymer was observed for HDPE/GC and HDPE/CNT composites. However, the decrease of ΔHm values for HDPE composites with graphite and graphene nanoplatelets relative to pure HDPE seems to be insignificant.

**Figure 3:**
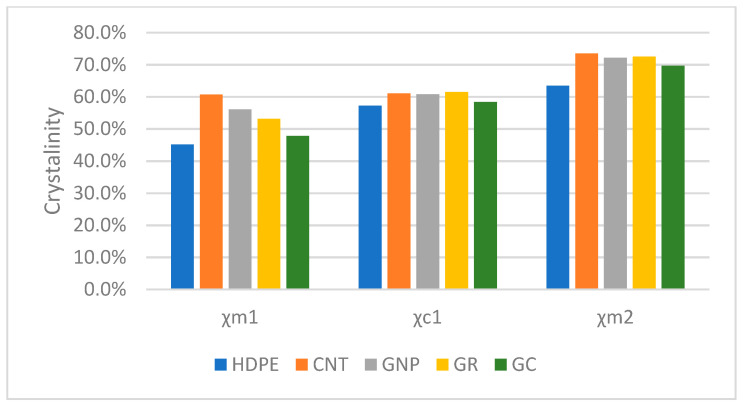
Crystallinity comparison of HDPE and HDPE/carbon composites. Χm1—crystallinity calculated from the first melting round, Χc1—crystallinity calculated from crystallization peak, Χm2—crystallinity calculated from the second melting round.

It is known that the degree of crystallinity, crystallites size, and crystal form significantly affect the mechanical properties of semi-crystalline thermoplastic matrices in composites. Therefore, understanding the effect of carbon filler particles on nucleation during the crystallization of such materials is crucial for determining structure-property relationships. The degree of crystallinity (X%) of pure HDPE and HDPE/carbon composites is presented in Figure 3.

The DSC results confirm changes in crystalline structure depending on the carbon filler type. All carbon reinforcements cause the crystallinity of HDPE to increase significantly from c.a. 62% to c.a. 70% because of heterogeneous nucleation of orthorhombic HDPE crystals onto the surface of carbon fillers. This result shows that glassy carbon has a similar impact oncrystallization in HDPE as Gr and CNT agglomerates. Even though GC consistsofdistorted graphene layers in structure, the size of the glassy carbon particles seems to be crucial for the crystallization of HDPE as no shifts in T_c,onset_ were observed.

Crystallites size in the HDPE/composite samples is expected to be much smaller than that of unfilled HDPE because of the similar crystallinity and numerous nucleating sites in all composites filled with various carbon particles. This was confirmed by XRD measurements (Figure 1, Table 1).

### 3.2. Hardness and Tensile Properties

**Figure 4:**
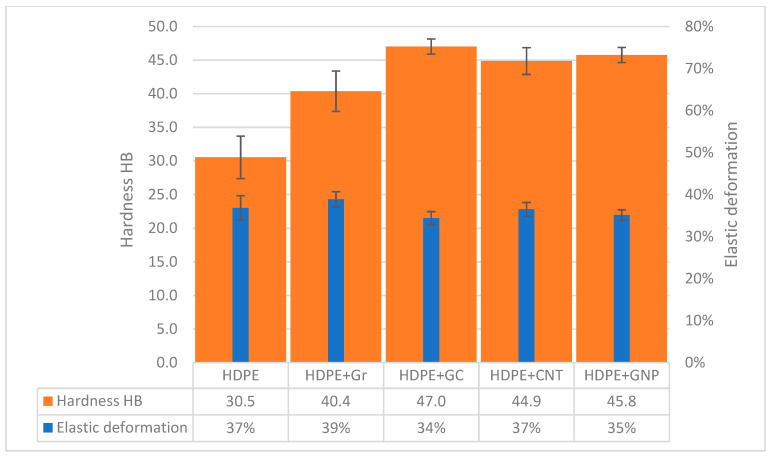
Comparison of Brinell hardness and elastic deformation of HDPE and HDPE carbon composites.

In Table 4, the results of static tensile testing are presented. The results show that GC increases tensile strength similarly to CNT and GNP. The glassy carbon and carbon nanotubes give similar increases in tensile strength (around 30%), while the addition of graphene results in a 50% improvement in tensile strength. Such increases in material strength were reported previously by others [34,35,36,37]. The increase in hardness is also visible (Figure 4). The toughest composite was the HDPE/GC composite. The observed increase is a result of higher crystallinity and synergic “cooperation” of the matrix with reinforcing particles, which is unseen in the HDPE/Gr composite. The described effect of GC onmechanical properties is a result of the reductionof crystallite size and residual stress. Comparing the results with reported data for CNT and GNP shows that glassy carbon can affect mechanical properties in a similar way.

## 4. Conclusions

Results from high-density polyethylene–glassy carbon investigations have been presented. The crystallinity was evaluated with two methods. Also, a novel approach to the analysis of residual stresses in the materials has been performed. The following conclusions can be drawn from the research:

1. Glassy carbon impacts crystallization and crystal structure similarly to graphene. However, the size of GC powder has a crucial impact at crystallization onset.
2. The GC particles in the HDPE matrix exhibit the smallest orthorhombic crystal lattice size and the smallest residual stress, in comparison with the other studied composites.
3. Proposed methods of residual stress determination (both, iso-inclination and extended Bragg’s law) obtain reliable and recurrable results and could be successfully applied to polymers and polymer matrix composites.
4. Addition of glassy carbon increased material strength due to reduced crystallite size and residual stress in the crystal phase. A similar effect was observed for composites with CNT and GNP.

The glassy carbon can be used successfully as a reinforcement for HDPE composite with properties like composites with carbon nanotubes and graphene.

## Figures and Tables

**Table 1:** Lattice parameters, crystallite size, lattice strain and d-spacing of examined materials.

Material	Lattice Parameters				Crystallite Size, nm	Lattice Strain	Crystallinity, %
	a, Å	b, Å	c, Å	V, Å^3^			
ICDD	4.94	7.24	2.54	93.2	-	-	-
Neat HDPE	4.97	7.45	2.56	94.6	79.8 ± 10.0	1.41 ± 0.02	48.4
HDPE + GC	4.93	7.41	2.55	93.1	32.1 ± 0.6	1.42 ± 0.04	49.2
HDPE + Gr	4.93	7.41	2.55	93.1	56.5 ± 4.3	1.33 ± 0.05	49.1
HDPE + GNP	4.93	7.41	2.55	93.0	37.5 ± 1.4	1.41 ± 0.03	49.8
HDPE + CNT	4.93	7.41	2.55	93.0	40.4 ± 1.8	1.45 ± 0.04	49.2

**Table 2:** Residual stress analysis was performed with two different methods for HDPE and HDPE composites.

Material	Residual Stress Analysis Using (020) Peak		Extended Bragg’s Law
	Linear Stress, MPa	Shear Stress, MPa	Stress, MPa
Neat HDPE	0.87 ± 0.46	0 ± 0	0.9 ± 0.06
HDPE + GC	0.28 ± 0.04	0.2 ± 0.03	0.2 ± 0.05
HDPE + Gr	−0.8 ± 0.26	0.2 ± 0.18	−0.87 ± 0.12
HDPE + GNP	0.6 ± 0.48	0.2 ± 0.10	0.45 ± +0.05
HDPE + CNT	−0.4 ± 0.27	1.8 ± 0.16	−1.15 ± 0.15

**Table 3:** Crystallization and melting parameters of HDPE and HDPE/carbon composites determined by DSC analysis. T_c,onset_ crystallization peak onset temperature; T_c,max_ crystallization peak maximum temperature; ΔHc, crystallization enthalpy with respect to the HDPE amount; T_m,max_ melting temperature; ΔH_m_, melting enthalpy (heat of fusion) normalized with respect to the HDPE amount.

Sample	T_c,onset_ (°C)	T_c,max_ (°C)	ΔH_c_ (J/g)	T_m,max_ (°C)	ΔH_m_ (J/g)
HDPE	116.2	113.3	174.7	131.5	210.3
HDPE + GC	115.1	111.9	168.9	132.3	201.8
HDPE + Gr	115.5	112.1	177.9	132.1	209.8
HDPE + GNP	120.6	116.2	176.5	132.7	209.5
HDPE + CNT	115.6	112.0	171.1	132.5	197.3

**Table 4:** Tensile strength results of the composites.

Material	1	2	3	Average
HDPE	13.43 MPa	11.32 MPa	12.15 MPa	12.30 ± 1.07 MPa
HDPE + GC	16.36 MPa	14.25 MPa	18.27 MPa	16.29 ± 2.01 MPa
HDPE + CNT	13.47 MPa	19.43 MPa	16.77 MPa	16.56 ± 2.99 MPa
HDPE + GNP	17.04 MPa	18.91 MPa	19.84 MPa	18.59 ± 1.43 MPa
HDPE + Gr	14.98 MPa	14.91 MPa	15.06 MPa	14.98 ± 0.08 MPa

## Data Availability

Not applicable.
